# Symptomatic Common Carotid Artery Stenosis With a Persistent Primitive Hypoglossal Artery Presenting With Posterior Circulation Symptoms and Technical Challenges in Stenting

**DOI:** 10.7759/cureus.81562

**Published:** 2025-04-01

**Authors:** Keisuke Kadooka, Roselyn Pamatmat, Kotaro Ueda, Shimpei Tsuboki, Takafumi Mitsutake, Michihiro Tanaka

**Affiliations:** 1 Department of Neuroendovascular Surgery, Kameda Medical Center, Kamogawa, JPN; 2 Department of Neurology, The Medical City South Luzon, Santa Rosa, PHL; 3 Department of Neurosurgery, Ariake Medical Center, Arao, JPN

**Keywords:** carotid artery stenosis, carotid artery stenting, embolic protection, persistent primitive hypoglossal artery, posterior circulation

## Abstract

The persistent primitive hypoglossal artery (PPHA) is a rare variant of the persistent carotid-vertebrobasilar anastomoses. When PPHA coexists with carotid artery stenoses, it typically presents ischemic symptoms of the anterior circulation. However, we report a unique case of common carotid artery (CCA) stenosis with PPHA presenting exclusively ischemic symptoms of the posterior circulation, which posed significant diagnostic challenges and required innovative modifications in embolic protection strategies during carotid artery stenting.

A 65-year-old woman experienced recurrent bilateral ptosis, diplopia, and transient bilateral visual loss, suggestive of posterior circulation ischemia. Imaging revealed significant left CCA stenosis with a PPHA supplying the posterior circulation. Due to the large diameter of the CCA, standard distal protection was unfeasible. Instead, distal balloon protection was innovatively applied at the bifurcation of the PPHA and the internal carotid artery, where the slightly narrower diameter facilitated successful stenting. The postoperative course was favorable, with no recurrence of symptoms.

PPHA-associated carotid stenosis can cause posterior circulation symptoms, complicating diagnosis. Understanding anatomical and hemodynamic variations like PPHA is crucial for effective treatment and ensuring optimal outcomes.

## Introduction

Persistent primitive hypoglossal artery (PPHA) is one of the persistent carotid-vertebrobasilar anastomoses and a rare embryological remnant, with a reported detection rate ranging from 0.027% to 0.29% [1,2]. Other known carotid-vertebrobasilar anastomoses include the persistent primitive trigeminal artery, the persistent primitive otic artery, and the proatlantal artery types I and II, although the existence of the persistent primitive otic artery remains controversial [3]. Among these anastomoses, the PPHA is regarded as the second most common carotid-vertebrobasilar anastomosis, following the persistent primitive trigeminal artery (ranging from 0.5% to 0.7%) [4,5] in frequency.

The PPHA is an anastomosis between the internal carotid artery (ICA) and the vertebral artery. Several reports have described its association with carotid artery stenosis and aneurysms [6-12]. In most reported cases of carotid artery stenosis involving a PPHA, patients typically present with symptoms related to the anterior circulation, similar to conventional carotid artery stenosis, or a combination of both anterior and posterior circulation symptoms [6-9].

Here, we present a rare case of common carotid artery (CCA) stenosis associated with a PPHA. The patient presented exclusively with posterior circulation ischemic symptoms, an atypical presentation that differs from both typical carotid artery stenosis and previously reported PPHA-associated cases. Initially, the case was not diagnosed as a transient ischemic attack or ischemic stroke, but was instead considered to be due to a nonneurological condition or of unknown origin, presenting a diagnostic challenge.

The patient was treated with carotid artery stenting (CAS), which required special consideration for embolic protection, as both the ICA and the PPHA needed to be protected. This case highlights two clinically important points: first, that carotid artery stenosis, typically associated with anterior circulation symptoms, can in rare instances present solely with posterior circulation ischemia, potentially delaying diagnosis; and second, that CAS under these circumstances requires strategies to protect both the anterior and posterior circulations. We describe a feasible technique to achieve this.

## Case presentation

A 65-year-old Japanese woman was previously independent in activities of daily living with a modified Rankin Scale score of 0. Her medical history included right-sided breast cancer (treated with surgery six years ago at stage I and currently in remission), *Helicobacter pylori* infection (eradicated), hypertension (under treatment), and dyslipidemia (under treatment). She was a nondrinker but had a 40-pack-year history of smoking. She had no prior history of stroke or cardiovascular disease.

Two weeks before visiting our general physician, she developed transient episodes of bilateral ptosis with difficulty opening her eyes, diplopia, transient bilateral visual loss (each episode lasting less than five minutes), and dizziness. She was asymptomatic at the time of examination, but due to the ptosis episodes, myasthenia gravis was initially suspected, and she was referred to the neurology department two weeks later. She had no history of gross motor weakness or sensory disturbances. At the neurology consultation, she was again asymptomatic, and a decision was made for follow-up observation. However, three days after the consultation, she experienced a single episode of transient visual loss in her left eye and revisited the neurology department. This led to a suspicion of amaurosis fugax and carotid artery stenosis, prompting carotid ultrasound and brain MRI. Blood tests revealed mild elevations in C-reactive protein, fasting blood glucose, and the low-density lipoprotein/high-density lipoprotein cholesterol ratio. The key blood test results are shown in Table 1.

**Table 1:** The key blood test results HDL: high-density lipoprotein; LDL: low-density lipoprotein; PG: plasma glucose; HbA1c: hemoglobin A1c; NGSP: National Glycohemoglobin Standardization Program; INR: international normalized ratio

Parameter (unit)	Value	Reference range
Blood urea nitrogen (mg/dL)	15	8-22
Creatinine (mg/dL)	0.69	0.6-1.2
Estimated glomerular filtration rate (mL/minute/1.73 m^2^)	64.93	-
Aspartate aminotransferase (U/L)	26	13-33
Alanine aminotransferase (U/L)	21	8-42
Gamma-glutamyl transferase (U/L)	14	10-47
Total protein (g/dL)	7.5	6.7-8.3
Albumin (g/dL)	4.2	3.4-5.8
C-reactive protein (mg/dL)	0.6	0-0.14
Triglycerides (mg/dL)	81	50-149
Total cholesterol (mg/dL)	139	150-219
HDL cholesterol (mg/dL)	34	40-70
LDL cholesterol (mg/dL)	88	70-139
L/H ratio	2.59	-
Blood glucose (PG) (mg/dL)	124	70-110
HbA1c (NGSP) (%)	5.9	4.6-6.2
White blood cell count (10^2^/μL)	66	35-98
Red blood cell count (10^4^/μL)	399	370-500
Hemoglobin (g/dL)	12.2	11-15.3
Platelet count (10^4^/μL)	26.6	13-37
Prothrombin time (INR)	1	0.80-1.30
Activated partial thromboplastin time (seconds)	28.1	20-30
Fibrinogen (mg/dL)	520	150-500
Fibrin degradation products (μg/mL)	1.4	0.1-5
D-dimer (μg/mL)	0.1	0.1-0.5

**Figure 1:**
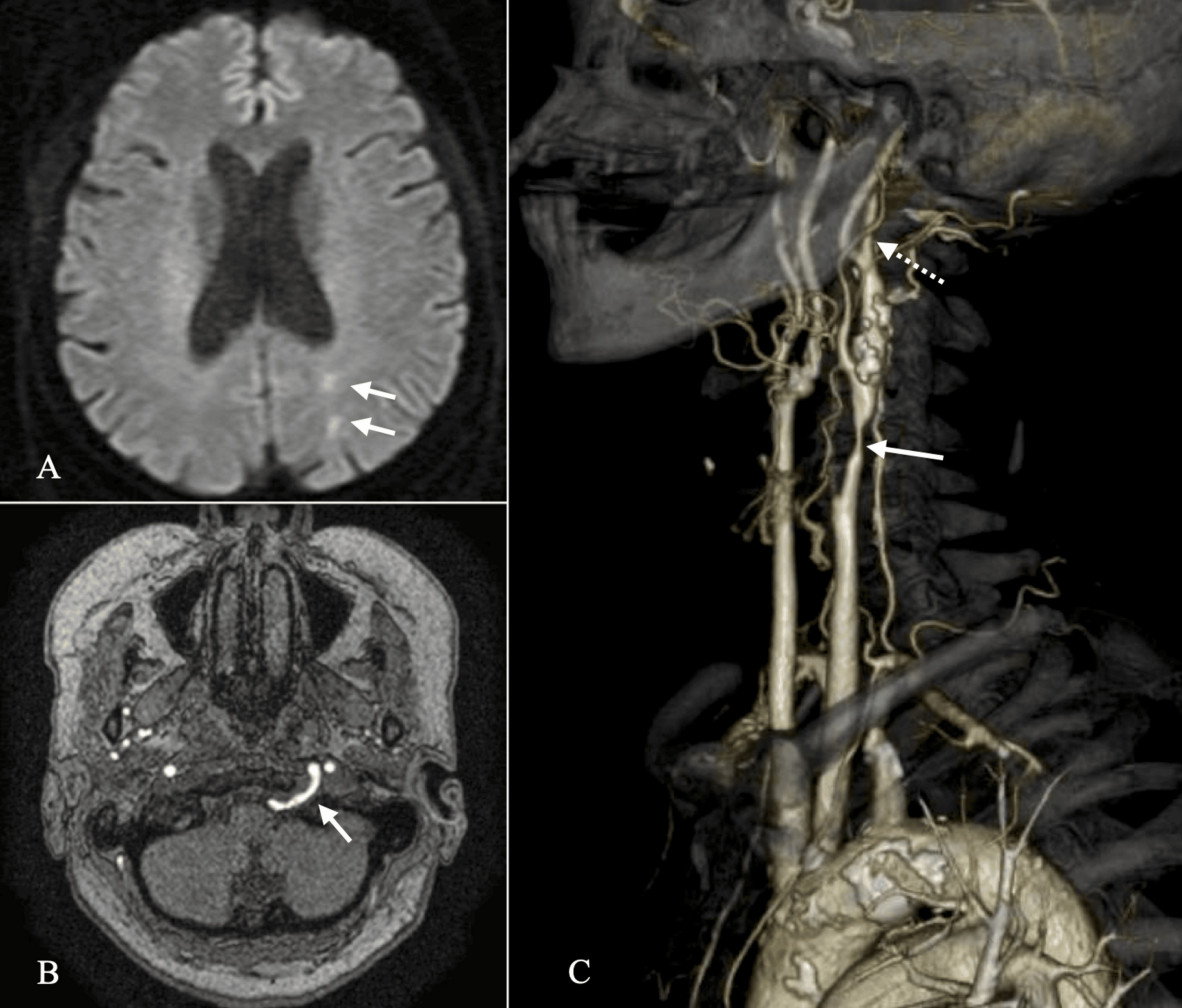
Preoperative MRI and CT angiography. (A) DWI showing hyperintensity at the left parietooccipital area (white arrows). (B) MR angiography showing PPHA running through the left hypoglossal canal (white arrow). (C) CT angiography revealing CCA stenosis (white arrow). PPHA originates from the ICA at the level of the mandibular angle (white dotted arrow) DWI: diffusion-weighted imaging; PPHA: persistent primitive hypoglossal artery; CCA: common carotid artery

Initial diffusion-weighted imaging revealed spot lesions in the left parietal lobe, located within the middle cerebral artery-posterior cerebral artery borderzone at the level of the lateral ventricular body, which appeared unrelated to her symptoms (Figure 1). Carotid Doppler ultrasonography revealed severe left CCA stenosis with 96% luminal narrowing and a maximum flow velocity (Vmax) of 500 cm/second.

Based on these findings, symptomatic carotid artery stenosis was diagnosed, and she was referred to our department. However, it took one month from symptom onset to diagnosis. Additionally, CT angiography and MR angiography demonstrated significant CCA stenosis and the presence of a PPHA originating from the left ICA, which served as the primary perfusion route to the vertebrobasilar circulation (Figure 1).

The symptoms preceding amaurosis fugax, including bilateral ptosis, diplopia, transient bilateral visual loss, and dizziness, were atypical for ICA stenosis, which typically presents with anterior circulation symptoms. Instead, these symptoms were more consistent with posterior circulation involvement affecting the brainstem and bilateral occipital lobes. This atypical presentation contributed to the diagnostic delay. Given the high-grade symptomatic carotid stenosis, CAS was indicated.

**Figure 2:**
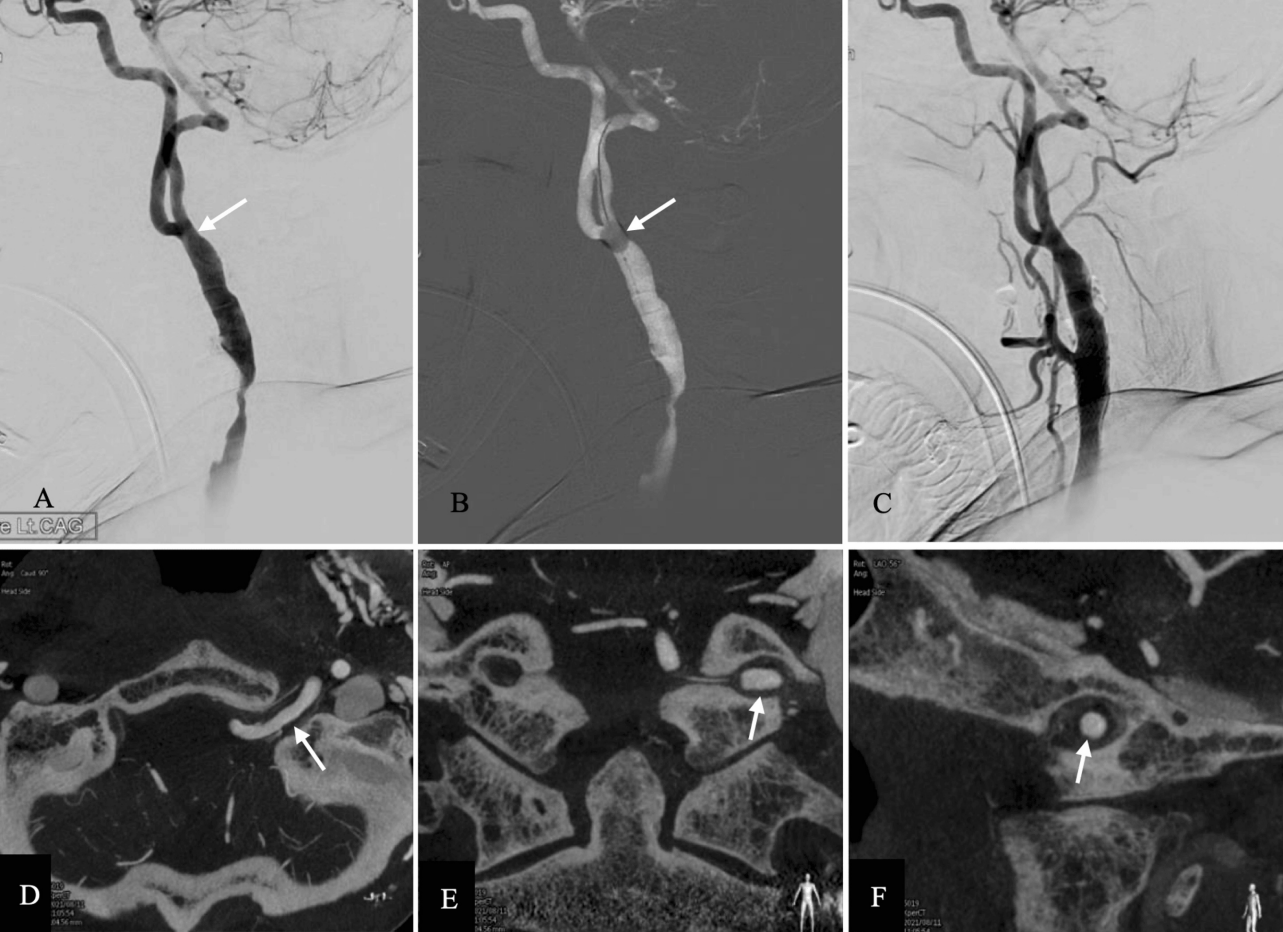
Treatment and cone beam CT of the PPHA. (A) Preoperative left CCA injection showing CCA stenosis. The caliber of the distal CCA is too large to occlude with a distal balloon protection device. Note the narrow segment at the bifurcation (white arrow). (B) Intraoperative roadmap. The distal balloon (white arrow) protection device is located at the ICA and PPHA bifurcation, where its diameter is small enough for the distal balloon protection devices. (C) Postoperative injection showing successful stenting. Cone beam CT revealing left PPHA (white arrows) running through the hypoglossal canal: (D) axial, (E) coronal, and (F) sagittal CT: computed tomography; PPHA: persistent primitive hypoglossal artery; CCA: common carotid artery

The patient underwent CAS under general anesthesia while on dual antiplatelet therapy. Vascular access and distal embolic protection were achieved using a 6-Fr guiding sheath (Axcelguide, Medikit, Tokyo, Japan) and a distal balloon protection device (PercuSurge GuardWire, Medtronic, Minneapolis, MN). During embolic protection, it was necessary to safeguard both the ICA and PPHA. However, the distal CCA diameter beyond the stenotic site exceeded 6 mm, preventing effective vessel occlusion with the PercuSurge GuardWire. Fortunately, a segment with a diameter of less than 6 mm was identified at the bifurcation of the ICA and PPHA, allowing effective occlusion at this site. This enabled simultaneous protection of both the ICA and PPHA using a single balloon (Figure 2). Predilatation was performed with a 5 x 30 mm balloon (Rx-Genity, KANEKA, Tokyo, Japan), followed by deployment of two overlapping stents (Precise 8 x 40 mm and 10 x 40 mm, KANEKA, Tokyo, Japan) to cover the stenosis. Finally, postdilation with an 8 x 20 mm balloon (RX-Genity) ensured optimal expansion and restoration of blood flow (Figure 2). Intraoperative cone-beam CT clearly visualized the PPHA (Figure 2).

The procedure was successful with no perioperative complications. Thereafter, the patient’s symptoms completely resolved. Antiplatelet therapy was completed after three months of dual antiplatelet therapy (aspirin 100 mg/day and clopidogrel 75 mg/day), followed by three months of aspirin monotherapy (100 mg/day). Follow-up imaging at the two-year mark showed no evidence of restenosis.

## Discussion

In this case report, we describe a patient with PPHA, a rare vascular variant classified among the carotid-vertebrobasilar anastomoses. As noted earlier, this group also includes the persistent primitive trigeminal artery, the persistent primitive otic artery (whose existence remains controversial) [3], and the proatlantal arteries (types I and II). Among these embryonic anastomoses, PPHA is considered the second most frequently observed, following the persistent primitive trigeminal artery, which has been reported in 0.5%-0.7% of cases [4,5].

Although some cases of carotid artery stenosis associated with PPHA have been reported [6-9], the present case demonstrated the following unique features compared to previously published cases. First, the stenosis in the CCA reduced the blood flow to the PPHA, which exclusively supplied the posterior circulation. This led to multiple early symptoms of posterior circulation ischemia, including bilateral ptosis, diplopia, transient bilateral visual loss, and dizziness. The absence of anterior circulation symptoms until the onset of amaurosis fugax contributed to a delayed diagnosis, as typical carotid stenosis was not initially suspected. Second, regarding embolic protection, it was necessary to protect both the ICA and the PPHA, which connect to the posterior circulation. This required innovative approaches.

Neurologically and therapeutically, these features are exceedingly rare. Concerning the first point, a review of reported cases of PPHA-related carotid artery stenosis revealed that most cases present with symptoms of anterior circulation [6,7]. Cases presenting solely with posterior circulation symptoms are extremely rare and generally limited to dizziness [8,9]. As far as we know, no cases presenting with symptoms similar to those in this case (bilateral ptosis with difficulty opening the eyes, diplopia, and transient bilateral visual loss) have been reported.

Several differential diagnoses may be considered to partially account for the patient’s symptoms, including myasthenia gravis (causing transient bilateral ptosis), seizure or migraine, and posterior reversible encephalopathy syndrome, all of which have been associated with transient bilateral visual loss. However, hemodynamic compromise in the posterior circulation due to CCA stenosis associated with PPHA provides a more comprehensive and unifying explanation for the constellation of clinical findings observed in this case.

Recognizing that CCA stenosis with PPHA can present differently from typical carotid stenosis is important. Another notable carotid-vertebrobasilar anastomosis is PPTA, which has been reported in a case report to cause brainstem infarction in the presence of carotid artery stenosis [13].

Regarding the second point, various protection devices have been utilized based on anatomical characteristics [6-9]. Strategies include proximal protection [6], placement of filter devices for both ICA and PPHA [7], and a case where, unlike in this case, the stenosis was not in the CCA but in a narrower ICA, allowing for conventional distal balloon protection [9]. In the present case, stenosis was in the CCA. Although distal balloon protection was attempted, the large diameter of the CCA prevented effective occlusion. Therefore, a novel approach was employed by positioning the balloon at the bifurcation of the ICA and PPHA, protecting both arteries with a single balloon. This technique has not been previously reported. This technique can be performed using a single protection device and does not carry the risk of retrograde flow from the external carotid artery associated with proximal protection. If the vessel diameter at the ICA-PPHA bifurcation allows for the placement of a distal balloon, this approach may represent an optimal strategy.

A key limitation of this report is the small sample size, as it is based on a single case. It remains uncertain whether the findings can be generalized to other cases of carotid artery stenosis associated with PPHA, particularly with respect to the presentation of isolated posterior circulation ischemic symptoms and the suitability of the ICA diameter proximal to the PPHA origin for distal balloon placement.

## Conclusions

Posterior circulation ischemic symptoms can occur in cases of carotid artery stenosis when associated with PPHA, making diagnosis particularly challenging. Treating CCA stenosis with PPHA needs special consideration. Distal balloon protection at the level of the bifurcation of the ICA and PPHA could effectively protect both arteries. Understanding the anatomical variations of the carotid-vertebrobasilar anastomoses, such as PPHA, is crucial in both diagnosing atypical ischemic presentations and planning endovascular treatments to ensure optimal outcomes.
